# Postural stability and muscle co-activation among younger adults adapting to an immersive virtual reality

**DOI:** 10.1007/s00421-026-06177-x

**Published:** 2026-03-02

**Authors:** Ulrik Röijezon, Eva Ekvall Hansson, Jenny Älmqvist Nae, Elin Östlind, Per-Anders Fransson

**Affiliations:** 1 Department of Health Sciences, Lund University, Lund, Sweden; 2 Department of Health, Education and Technology, Luleå University of Technology, Luleå, Sweden; 3 Department of Otorhinolaryngology Head and Neck Surgery, Skåne University Hospital, Lund, Sweden; 4 Department of Clinical Sciences Lund, Lund University, Lund, Sweden

**Keywords:** Adaptation, Immersive visual environments, Muscle activity, Postural stability, Sensorimotor rehabilitation, Virtual reality

## Abstract

Immersive visual environment can deceive our interoceptive perception of how we move relative to our environment. If the visual environment or a high reliance on vision produces straining work conditions, motion sickness or is associated with a medical disorder, the causes merit addressing. Therefore, the purpose of this study was to investigate whether postural control adapts to challenging visual environments from VR by adjusting postural stability and muscle co-contractions responses and investigate associations between stability measures and postural muscle co-contractions during VR-stimulation and control tests in quiet stance. Twenty-eight young adults participated (mean age 25.3 years, SD 4.6 years). In five sessions, they watched a VR simulation of a roller coast ride while a force platform recorded postural stability and electromyography recorded muscle co-contractions in leg muscles. Two control tests (quiet stance with eyes open and eyes closed), were performed before the VR sessions. The first VR session significantly increased the postural stability energy (*p* ≤ 0.002) and the muscle co-contraction levels (*p* ≤ 0.008) compared with the eyes open control test. Repeated VR sessions produced an adaptation that decreased postural stability energy (*p* ≤ 0.033) and muscle co-contraction levels (*p* ≤ 0.028). VR-stimulation made the association between postural stability responses and muscle co-contractions more prominent. Immersive visual environments can produce marked challenges to postural control, but the repeated experiences can also initiate sensory reweighting increasing resilience to visual disturbance.

## Introduction

Postural control mechanisms maintain balance, even under challenging conditions (Shumway-Cook 2008). Several sensory systems are involved and interact with the central nervous system to send adequate motor commands to the postural muscles (Shumway-Cook 2008). When responding to a disturbance in balance, constant information from the sensory systems enables ongoing corrections and reactions, known as feedback control. In contrast, feedforward control occurs when the system can plan and prepare for postural actions based on experience of similar situations. Another strategy, especially during novel or unpredictable tasks, is to increase neuromuscular stiffness around the joints, i.e., stiffness control (Franklin and Wolpert 2011).

Most of us have experienced - e.g., when sitting on a train at a train station, or more deliberately from theme park events - that immersive visual environment can deceive our interoceptive perception of how we move relative to our environment. As isolated events, this usually causes no major problem for us. However, if the visual environment or a high reliance on vision produces continuously straining work conditions, cause motion sickness or if medical disorders produce distorted visual information, the causes merit addressing.

Several medical disorders can cause disturbances in the interactions between the sensory systems and the central nervous system, and lead to a sensory mismatch, e.g., acute vestibular loss (Cullen et al. 2009) or motion sickness (Laessoe et al. 2023). Vision is one of the sensory systems involved in maintaining balance in both younger (Gaerlan et al. 2012) and older adults (Sloane et al. 1989). Multisensory dizziness (Kao et al. 2001; Strupp et al. 2023) and persistent postural-perceptual dizziness (PPPD) (Staab et al. 2017) are examples of conditions that denote an increased reliance on vision to maintain balance. A common consequence from peripheral vestibular lesions and various central disorders is a disturbed spatial orientation, which may be exacerbated by exposure to distorted visual environment (Bronstein 1995; Zwergal et al. 2024). This is of importance for the assessment and treatment of balance disorders, since these conditions may not be appropriately detected with standard clinical tests such as caloric irrigation, and thereby not treated with the most efficient interventions (Malmström et al. 2020).

Recent studies have suggested that Virtual Reality (VR) might address sensory mismatch issues from sensory malfunction and central disorders (Truijen et al. 2022) by instigating a sensory reweight calibration from vision to other systems (Fransson et al. 2019; Sana et al. 2023; Sulway and Whitney 2019). One effect of this reweighting is an increased resilience in postural stability to visual disturbance after repeated VR-sessions (Almqvist Nae et al. 2024; Fransson et al. 2019).

Measurements of postural control, i.e., posturography, are commonly assessed on a force platform measuring center of pressure (CoP) trajectory, thereby indirectly assessing postural sway (Era et al. 2006). This is a swift method to record very accurate measures of the reaction forces of human postural stability. Research has shown that different measures from the posturographic measures represent different mechanisms. For example, decomposing the sway into slow (< 0.1 Hz) and fast (> 0,1 Hz) components can reveal alterations in different mechanisms of postural control (Kristinsdottir et al. 2001; Patel et al. 2009a, b). The methods and cut offs used to decompose CoP or torque into slow and fast components vary, but it has been argued that the slow component is in the feedback (sensory) loop, while the fast component is related to the motor output, i.e., the controlling of the Centre of Mass (CoM) (Kiemel et al. 2006). By combining posturographic assessment with measures of the postural stabilizing muscles it would be possible to gain further knowledge about the human postural control during postural challenges, and adaptations to repeated perturbations. To our knowledge, this has not been done during postural challenges using visual perturbations via VR.

The aim was to investigate whether postural control among healthy young adults adapts to challenging visual environments from VR by adjusting muscle co-contractions and postural stability responses, and whether repeated exposures trigger an adaptation. A further aim was to investigate associations between postural stability measures and postural muscle co-contractions during control tests and VR-stimulation.

## Materials and methods

### Ethical consideration

The study was performed in accordance with the latest Declaration of Helsinki and received ethical approval from the Ethical Review Authority in Sweden (Dnr:2023-00353-01). All participants gave their written consent prior to participation.

### Participants

Thirty subjects were recruited to participate in the study, whereof 28 were included in the final analysis (17 males, 11 females, mean age 25.3 years (standard deviation (SD) 4.6 years), mean height 1.80 m (SD 0.11 m), mean weight 75.8 kg (SD 13.5 kg), mean BMI 23.4 (SD 2.8). Two participants were excluded due to technical issues with loading stored data. Participants were included if they were young adults (range 18–45 years), considered themselves healthy, and had no prior history of diseases that could affect their balance, e.g., a known history of vertigo or neurological diseases. The participants were screened for health disorders by using a custom-made questionnaire, e.g., whether they had reduced balance, prior injury in the lower extremity the last year. The questionnaire has been used in previous studies (Almqvist Nae et al. 2024).

The recruitment of participants was conducted through contacts with students at Lund university, friends and family. Those who accepted to participate in the study were referred to the Movement and Reality Laboratory (MoRe-lab) at Lund University, Sweden, for the testing.

### Electromyography and postural stability assessment equipment and EMG normalization data collection

**Fig. 1:**
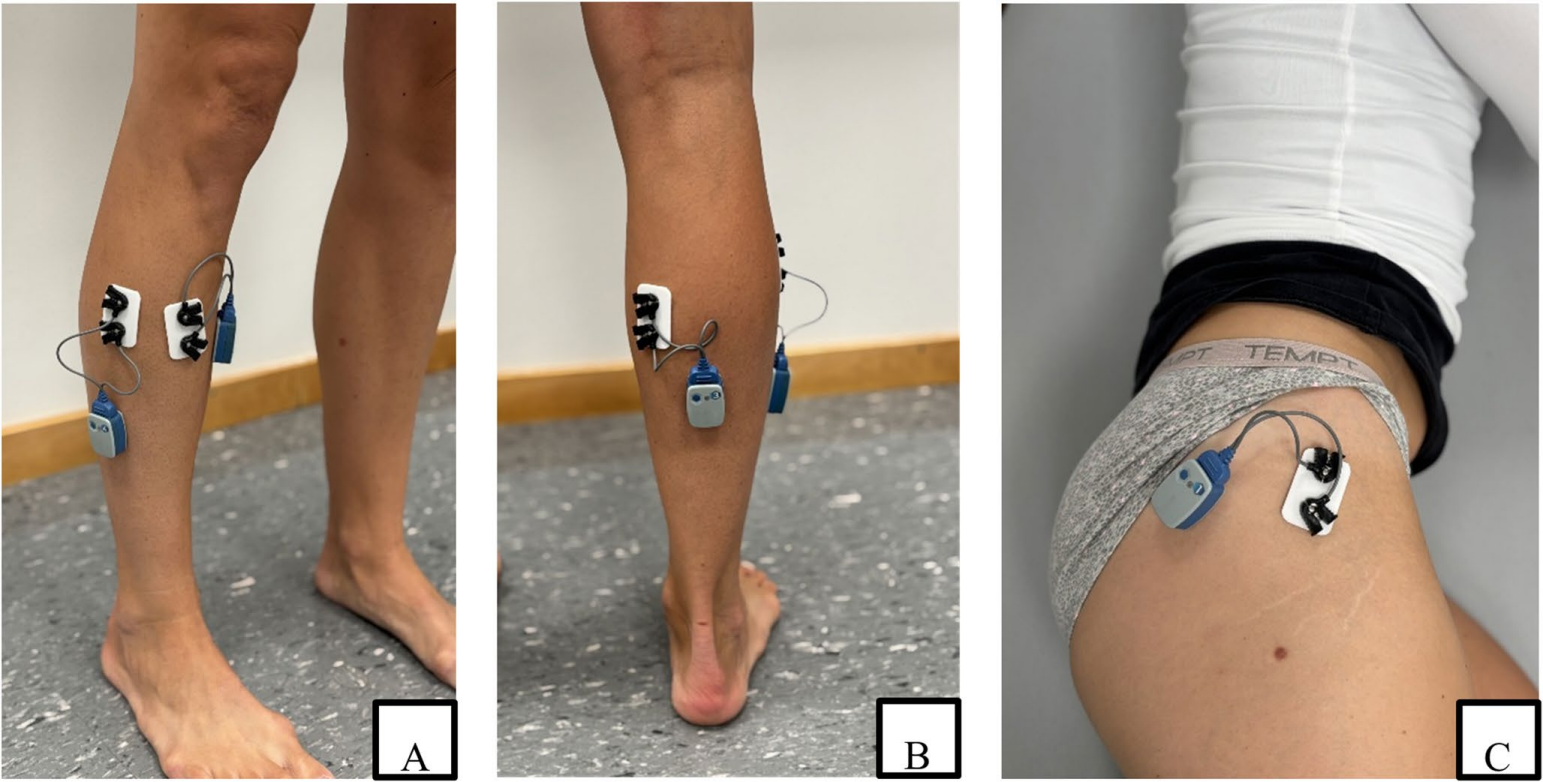
Electrode location for each muscle. **A** m tibialis anterior and m. peroneus longus, **B** m. gastrocnemius medial portion, **C** m. gluteus medius

The study hardware was designed to sample, synchronized in time, data at 1000 Hz from a posturography system and 2000 Hz from an electromyography (EMG) system. The posturography system was an AMTI force platform (AMTI HPS 464508, AMTI Europe GmbH, Helmstadt-Bargen, Germany). The EMG system was a wireless Noraxon Ultium system with 16 channels (USA. Inc, Scottdale, Arizona, USA), set to lowpass filter at 500 Hz sampled muscle activity before analysis. The skin was shaved and abraded using a medical abrasion gel (Nuprep, Weaver and Company, Aurora, Colorado, USA) before electrode placement. Dual surface electrodes with an inter-electrode distance of 20 mm (Noraxon, USA, Inc) were placed bilaterally on the postural muscles gluteus medius (GlutMed), medial gastrocnemius (MedGas), tibialis anterior (TibAnt), and peroneus longus (PerLong), according to the SENIAM (surface electromyography for non-invasive assessment of muscles) guidelines (Fig. 1 and Appendix 1) (Hermens et al. 2000).

Before performing study assessments, the maximum voluntary contraction (MVC) was assessed for each muscle, i.e., hip abduction for gluteus medius activation, plantar flexion for medial gastrocnemius activation, dorsi flexion for tibialis anterior activation, and pronation for peroneus longus activation. The procedure followed the SENIAM guidelines (Hermens et al. 2000), with a modification for gluteus medius MVC, which was performed in supine position instead of side-lying to prevent potential displacement or detachment of the bilaterally placed GlutMed electrode (Appendix 2). The MVC assessments were repeated three times for each muscle with 30 s pause between contractions. During each trial, subjects were instructed to gradually build up force and perform the contraction with maximal voluntary effort.

### Test procedure

The preparations and performance of all tests were executed on the same day and required approximately 90 min per person. The participants performed seven postural stability tests. Two control tests (quiet stance with eyes open, quiet stance with eyes closed), were performed in a randomized order before five VR sessions. During the control tests, the participants were instructed to stand once with their eyes closed and once focusing for two minutes on a target positioned at eye level on the wall 5 m in front of them. They were instructed to stand on a force platform without shoes and with their feet positioned with a slight outward rotation (approximately 30 degrees) using guidelines on the force platform, and thus, to adhere to a standardized posturography test protocol (Fransson et al. 2019; Patel, Fransson, et al., 2009; Patel et al. 2020). The participants were instructed to stand in a relaxed position, with their arms folded over their chest, and to keep their face straight forward. The control tests were used for assessing the normal postural sway and to provide a relative reference for the stability recorded when the participants were exposed to distortive visual information from VR. The participants did not wear the VR headset when performing the control tests since the VR equipment precluded proper visual feedback.

Before performing the five VR sessions, participants were shown a winter landscape 360° video while sitting. This was done to calibrate the sharpness of the images displayed through the VR visor before performing the postural stability assessments. The participants were then instructed to stand in the same way as during the control tests, on a force platform. The Oculus Quest 2 VR headset (Facebook Technologies, LLC, Menlo Park, California, USA) used in the study tracks the head movements in 360-degree range, and thus, any change in head direction produced an identical direction change in the VR movie displayed to the subject. Performing substantial head movements in a VR environment may produce additional strain for the participants. Hence, to make the test conditions standardized and as similar as possible for all subjects, the participants were instructed to look straight ahead during the VR sessions. A Quest 2 VR headset weighs about 503 g, which corresponds to the weight of a standard construction helmet.

Five postural stability assessments were thereafter made while exposed to the VR stimuli, repeatedly watching a photographic 360° video of “a roller-coast ride”, where the ride included numerous quick turns to the right and left and ascending/descending elevations. The same video was shown for 120 s on each session, with a 10-minute rest period between sessions. The participants were allowed to sit down and rest between repeated VR sessions. Illustrations of the kind of postural control responses evoked by watching an immersive VR movie can be found in the paper Fransson et al. 2019 (Fransson et al. 2019).

### Posturography analysis

The stability in anteroposterior and lateral directions during the control tests and repeated VR stimuli were determined by analyzing the variance of anteroposterior and lateral torque values from the force platform recordings. The torque variance values reflect the energy used towards the force platform to maintain stability (Johansson et al. 2009; Riccio and Stoffregen 1988). Moreover, biomechanical models of the human postural control system reveal that force platform recordings are directly influenced by two anthropometrical factors (i.e., the test subject’s weight and height) (Fransson 2009; Johansson et al. 2009). To appropriately address biases from these two anthropometrical effects, recorded torque values was regarded to reflect postural stability first after they had been normalized using the participants’ squared height and squared weight (Fransson et al. 2019; Johansson et al. 2009). The squared components in the variance formula made it necessary to normalize with squared parameters to achieve unit agreement.

In addition, spectral separations were performed to describe the smooth corrective changes of posture (i.e. low frequency energy < 0.1 Hz) and the fast corrective movements to maintain balance (i.e. high frequency energy > 0.1 Hz) (Kristinsdottir et al. 2001), using a fifth-order digital Finite duration Impulse Response (FIR) filter.

### EMG analysis

EMG data for the test conditions and the MVC assessments of GlutMed, MedGas, TibAnt and PerLong muscles were first processed from artifacts (Karacan et al. 2023) by using a bandpass filter with cutoffs at 20 Hz and 500 Hz and then calculating the mean root square (RMS) envelope using a sliding window of 75 ms. The MVC values were thereafter obtained by filtering the EMG recordings, using a moving average filter with a sliding window of 1 s, and then selecting the highest value in the resulting data. The EMG activity during the different test conditions was attained by normalizing the EMG for each muscle during test conditions by dividing the filtered and RMS averaged data with the corresponding MVC values. The co-contraction index (CCI) was calculated on sample level for the three muscle pairs: (1) gluteus medius right and left side (GlutMed L vs. GlutMed R), (2) tibialis anterior and medial gastrocnemius (TibAnt vs. MedGas), and (3) tibialis anterior and peroneus longus (TibAnt vs. PerLong). For each muscle pair, the muscle with the highest normalized EMG activity was regarded as agonist. CCI was computed using Rudolph’s method (Rudolph et al. 2001) with the normalized EMG amplitude values:

$$\:CCI=\:\frac{antagonist}{agonist}\left(antagonist+agonist\right)$$

In the statistical analysis, the mean and SD values for the CCI values were evaluated. These values were first calculated individually for each leg, Thereafter, the mean values using data for both legs were calculated and analyzed. The MATLAB 2023a software (The Mathworks Inc, Natick, Massachusetts, USA) was used for performing mathematical analyses.

### Statistical analysis

A repeated measures General Linear Model (GLM) Analysis of Variance (ANOVA) analysis was used for evaluating the performance across repeated VR sessions. The independent main factor analyzed for VR sessions were: ‘Repetition’ (Session 1…5; degree of freedom (d.f.) 4)). In the analysis, the dependent variables were the log-transformed normalized total, low frequency, and high frequency torque variance values (posturography data) and the log-transformed mean and SD muscle CCIs (EMG data). The model parameter Repetition was a within-subjects factor.

The Wilcoxon matched-pairs signed-rank test (Exact sig. 2-tailed) was used for post hoc within-subjects analyses of the accumulated effects of repeatedly performing VR Sessions i.e., analyzing the adaptive changes between VR Session 1 and VR Session 5; the difference between VR Session 1 and the eyes open control test; the difference between VR Session 5 and the eyes open control test; and for determining the role of vision using data from the control tests (Patel et al. 2008).

A Spearman’s correlation was performed to establish whether associations existed between postural stability values and the CCIs. The correlations describing the characteristics during VR were based on pooling together the data collected from all 5 VR sessions. The correlations describing the characteristics during control conditions were based on pooling together the data collected from both control tests. In the correlation analyses *p* < 0.05 were considered significant and the R-values were evaluated according to Cohens thresholds: >0.1 = small, > 0.3 = moderate, > 0.5 = strong and > 0.7 = very strong correlation.

In the GLM ANOVA analysis, *p* < 0.05 was considered significant. In the post-hoc analyses, *p* < 0.025 was considered significant after Bonferroni correction of the within-subjects’ analyses of VR data and *p* < 0.05 was considered significant for the within-subjects’ analyses of control tests data. Non-parametric statistical methods were used in the post hoc tests as the Shapiro-Wilk test revealed that some datasets were not normally distributed, and that normal distribution could not be obtained by log-transformation (Altman 1991).

Sample size analyses of the posturography parameters revealed an effect size of 0.95 which shows that with the p-value set to 0.05 (2-tailed), our study requires 11 subjects to reach a power value of 0.8 for the parameter used. The statistical analyses were performed using SPSS (Version 28, IBM Corp, Armonk, NY, USA) and the power analysis was performed with GPower 3.1.9.7.

## Results

### Posturography assessments

#### Influence of repetition on postural stability during VR sessions

Repeated measures GLM ANOVA analyses revealed that when participants repeatedly watched the same VR movie, the energy used within all spectral ranges in anteroposterior directions gradually decreased significantly (*p* ≤ 0.033), but not in lateral direction (Table 1).

**Table 1:** Influence of repetition on postural stability during VR sessions

VR stability^a^	Repetition^b^	
	Anteroposterior	Lateral
Total	**0.001 [13.0]**	0.182 [1.9]
Low < 0.1 Hz	**0.033 [5.1]**	0.267 [1.3]
High > 0.1 Hz	**< 0.001 [28.8]**	0.117 [2.6]

^a^Repeated measures GLM ANOVA analyses of how the postural stability during VR was affected by main factor “Repetition”

^b^Presented as: p-values and [F-values]. The notation “< 0.001” means that the p-value is smaller than 0.001. Found significant effects are marked in bold text

Significant differences are shown in bold figures

#### Post hoc evaluation of the initial effects and of adaptation to VR on postural stability

**Fig. 2:**
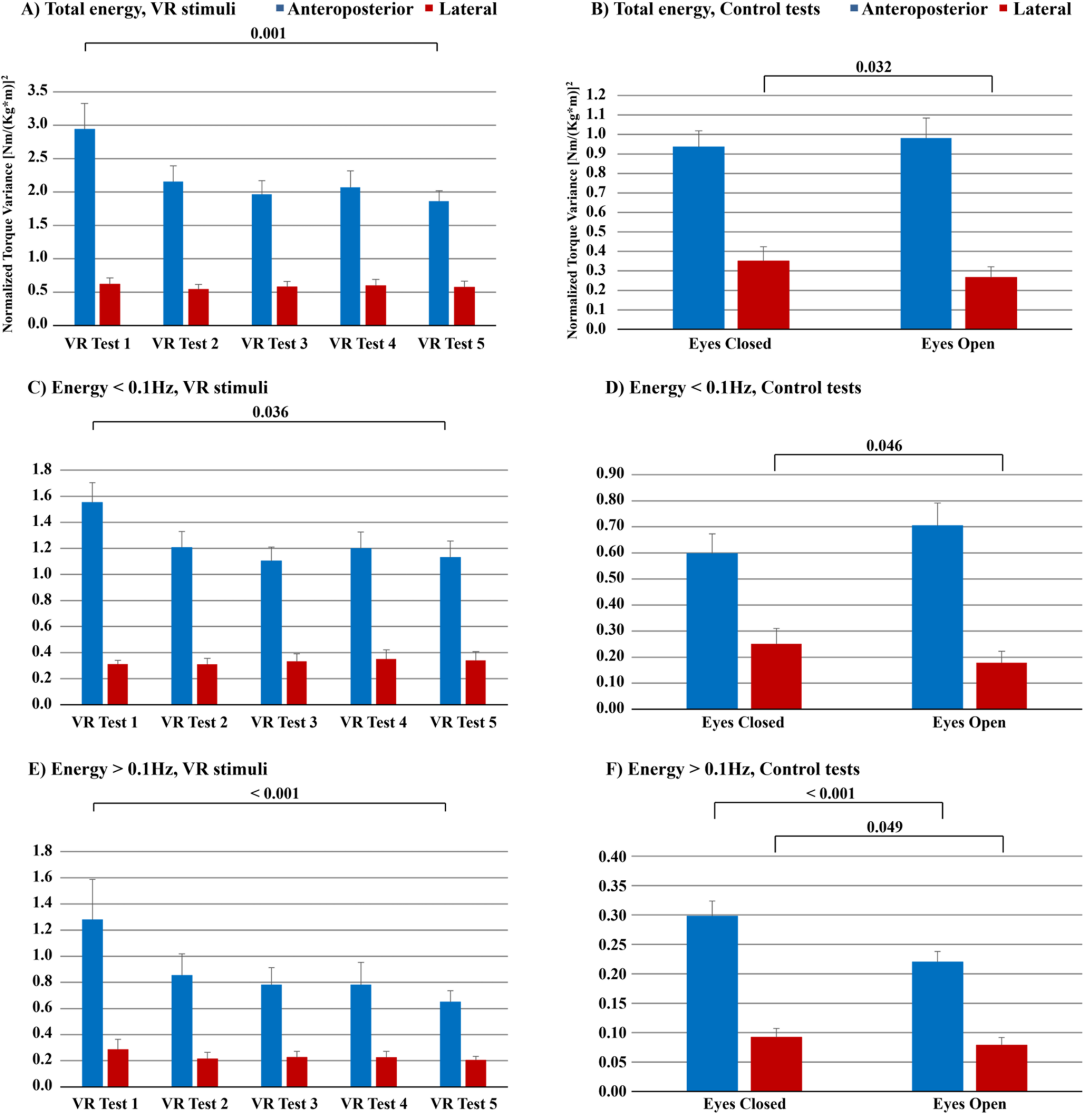
Postural stability during repeated VR sessions and during the control tests. The performance recorded during the five repeated VR sessions are presented as bars (mean) and whiskers (SEM) values of normalized total (**A**), low frequency (**C**) and high frequency (**E**) torque variance. The performance during the eyes closed and eyes open control tests are presented as normalized total (**B**), low frequency (**D**) and high frequency (**F**) torque variance

Within-subjects analyses revealed that participants used significantly more energy during VR session 1 vs. the control test with eyes open, in both anteroposterior (*p* < 0.001) and lateral (*p* ≤ 0.001) directions (Fig. 2; Table 2). In anteroposterior direction, total energy used was 200% higher; low frequency energy was 120% higher, and high frequency energy was 481% higher. In lateral direction, total energy used was 133% higher; low frequency energy was 74% higher and high frequency energy was 263% higher.

**Table 2:** Influence of initial VR and effects of adaptation on postural stability

Stability changes^a, b^	*p*-value	VR 1/EO^c^	*p*-value	VR 5/VR 1^d^	*p*-value	VR 5/EO^e^
Anteroposterior						
Total	**< 0.001**	**3.00 (1.19)**	**0.001**	**0.63 (0.07)**	**< 0.001**	**1.90 (0.30)**
Low < 0.1 Hz	**< 0.001**	**2.20 (0.84)**	0.036	0.73 (0.12)	**< 0.001**	**1.60 (0.18)**
High > 0.1 Hz	**< 0.001**	**5.81 (1.40)**	**< 0.001**	**0.51 (0.07)**	**< 0.001**	**2.96 (0.38)**
Lateral						
Total	**< 0.001**	**2.33 (0.57)**	0.272	0.93 (0.15)	**< 0.001**	**2.16 (0.29)**
Low < 0.1 Hz	**0.001**	**1.74 (0.66)**	0.303	1.10 (0.22)	**0.006**	**1.91 (0.43)**
High > 0.1 Hz	**< 0.001**	**3.63 (1.24)**	0.255	0.72 (0.19)	**< 0.001**	**2.61 (0.44)**

^a^Quotient values are presented as: group mean quotient (individual quotient SEM)

^b^A *p* < 0.025 was considered significant after Bonferroni correction

^c^Quotient values between VR session 1/control test with eyes open

^d^Quotient values between VR session 5/VR session 1

^e^Quotient values between VR session 5/control test with eyes open

Significant differences are shown in bold figures

Moreover, participants significantly reduced the energy used across the five VR sessions (i.e., energy used during VR session 1 vs. during VR session 5) in anteroposterior directions (*p* ≤ 0.001) (Table 2). In anteroposterior direction, total energy was reduced by 37% and high frequency energy was reduced by 49%.

There was, however, still a significantly higher energy use in VR session 5 compared to the control test with eyes open in both anteroposterior (*p* < 0.001) and lateral (*p* ≤ 0.006) directions (Table 2). In anteroposterior direction, total energy used was 90% higher; low frequency energy was 60% higher and high frequency energy was 196% higher. In lateral direction, total energy was 116% higher during VR; low frequency energy was 91% higher, and high frequency energy was 161% higher.

### Influence of vision on postural stability during the control tests

Within-subjects analyses revealed that the high frequency energy used in anteroposterior direction was significantly higher with eyes closed than with eyes open by 36% (*p* < 0.001) (Fig. 2; Table 3). In lateral direction, the total energy used was 31% higher, the low frequency energy was 40% higher, and the high frequency energy was 17% higher with eyes closed than with eyes open (*p* ≤ 0.049).

**Table 3:** Influence of Vision on control test postural stability

Stability changes^a, b^	*p*-value	EC/EO ^c^
Anteroposterior		
Total	0.582	0.95 (0.18)
Low < 0.1 Hz	0.218	0.85 (0.26)
High > 0.1 Hz	**< 0.001**	**1.36 (0.10)**
Lateral		
Total	**0.032**	**1.31 (0.18)**
Low < 0.1 Hz	**0.046**	**1.40 (0.38)**
High > 0.1 Hz	**0.049**	**1.17 (0.09)**

^a^Quotient values are presented as: group mean quotient (individual quotient SEM)

^b^A *p* < 0.05 was considered significant after Bonferroni correction

^c^Quotient values between control test with eyes closed / control test with eyes open

Significant differences are shown in bold figures

#### EMG assessments

##### Influence of repetition on CCI during VR sessions

**Fig. 3:**
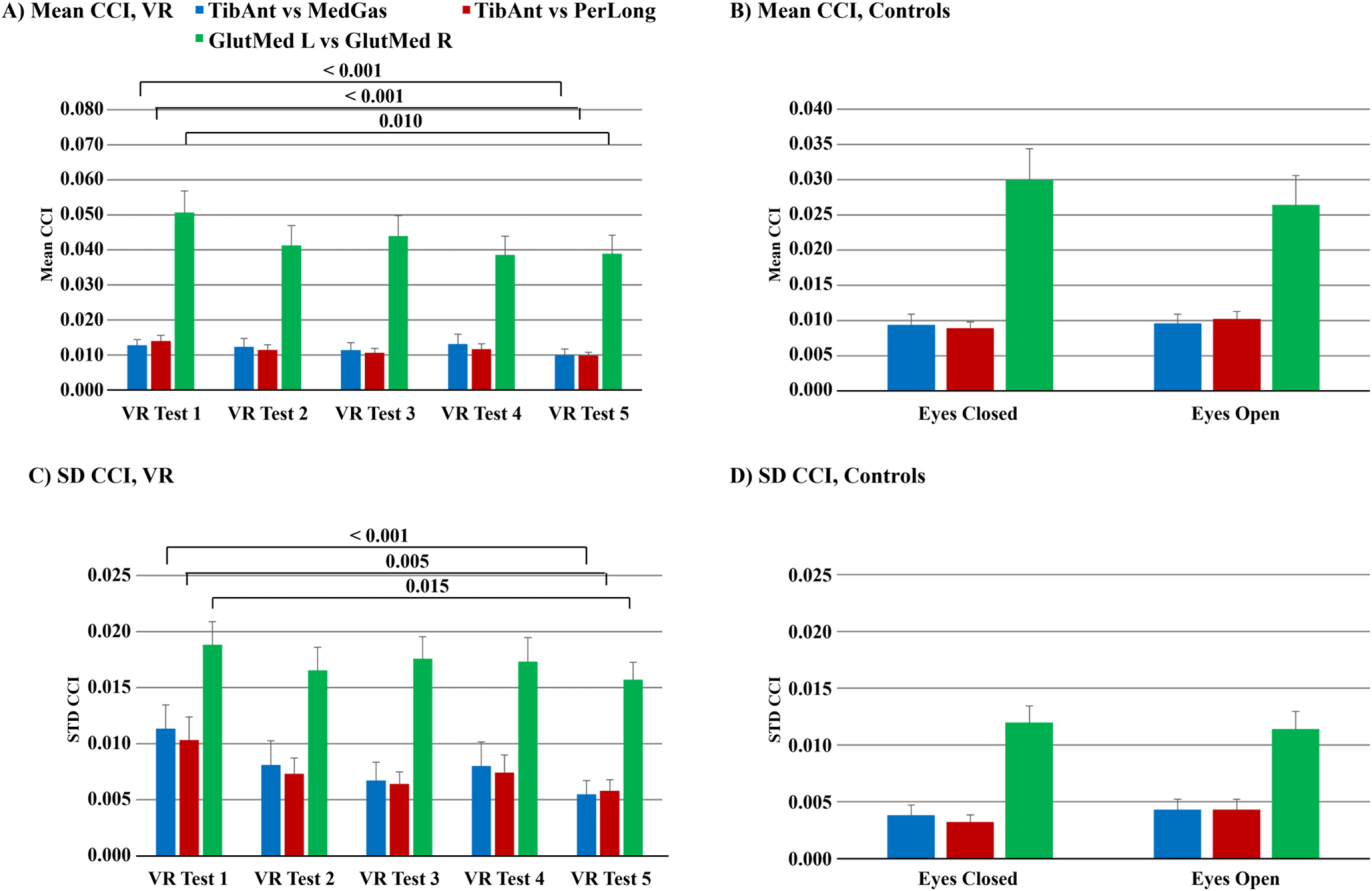
CCIs during repeated VR sessions and during the control tests. The CCIs between three muscle compositions during the five repeated VR sessions are presented as bars (mean) and whiskers (SEM) values of mean CCIs (**A**) and SD CCIs (**C**). The performance during the control tests with eyes closed and eyes open are presented as mean CCIs (**B**) and SD CCIs (**D**)

Repeated measures GLM ANOVA analyses revealed that mean (*p* ≤ 0.028) and SD (*p* ≤ 0.040) CCIs for the three muscle compositions TibAnt vs. MedGas, TibAnt vs. PerLong and GlutMed L vs. GlutMed R decreased significantly during the five VR sessions (Fig. 3; Table 4).

**Table 4:** Influence of repetition on CCI during VR sessions

CCI^a^	Repetition^b^	
	Mean	SD
TibAnt vs. MedGas	< 0.001 [20.9]	0.001 [13.4]
TibAnt vs. PerLong	0.004 [10.2]	0.040 [4.7]
GlutMed L vs. GlutMed R	0.028 [5.4]	0.006 [8.9]

^a^Repeated measures GLM ANOVA analyses of how the muscle co-contraction during VR was affected by main factor “Repetition”

^b^p-values and [F-values]

##### Post hoc evaluation of the initial effects and of adaptation to VR on CCI

Within-subjects analyses revealed that participants significantly increased the mean (*p* ≤ 0.002) and SD (*p* < 0.001) CCI used during VR session 1 vs. the control test with eyes open, between the three muscle compositions investigated (Fig. 3; Table 5). Mean CCI increased 33% for TibAnt vs. MedGas; increased 36% for TibAnt vs. PerLong and increased 92% for GlutMed L vs. GlutMed R. SD CCI increased 163% for TibAnt vs. MedGas; increased 139% for TibAnt vs. PerLong, and increased 65% for GlutMed L vs. GlutMed R.

**Table 5:** Influence of initial VR and effects of adaptation on CCI

CCI^a, b^	*p*-value	VR 1/EO^c^	*p*-value	VR 5/VR 1^d^	*p*-value	VR 5/EO^e^
Mean						
TibAnt vs. MedGas	**< 0.001**	**1.33 (0.14)**	**< 0.001**	**0.78 (0.05)**	0.171	1.04 (0.07)
TibAnt vs. PerLong	**0.002**	**1.36 (0.15)**	**< 0.001**	**0.70 (0.08)**	0.400	0.96 (0.06)
GlutMed L vs. GlutMed R	**< 0.001**	**1.92 (0.45)**	**0.010**	**0.77 (0.08)**	**< 0.001**	**1.47 (0.23)**
SD						
TibAnt vs. MedGas	**< 0.001**	**2.63 (1.09)**	**< 0.001**	**0.48 (0.13)**	**< 0.001**	**1.27 (0.16)**
TibAnt vs. PerLong	**< 0.001**	**2.39 (1.01)**	**0.005**	**0.56 (0.50)**	**< 0.001**	**1.34 (0.21)**
GlutMed L vs. GlutMed R	**< 0.001**	**1.65 (0.23)**	**0.015**	**0.83 (0.07)**	**< 0.001**	**1.38 (0.24)**

^a^Quotient values are presented as: group mean quotient (individual quotient SEM)

^b^A *p* < 0.025 was considered significant after Bonferroni correction

^c^Quotient values between VR session 1/control test with eyes open

^d^Quotient values between VR session 5/VR session 1

^e^Quotient values between VR session 5/control test with eyes open

Significant differences are shown in bold figures

Analyses performed to determine the effects of adaptation revealed that participants significantly reduced the mean (*p* ≤ 0.010) and SD (*p* ≤ 0.015) CCI across the five VR sessions for the three muscle compositions investigated (Table 5). Mean CCI decreased 22% for TibAnt vs. MedGas; decreased 30% for TibAnt vs. PerLong, and decreased 23% for GlutMed L vs. GlutMed R. SD CCIs decreased 52% for TibAnt vs. MedGas; decreased 44% for TibAnt vs. PerLong, and decreased 17% for GlutMed L vs. GlutMed R.

When comparing the mean and SD CCIs after adaptation, i.e., during VR session 5, with the values during the control test with eyes open, mean CCIs were still significantly 47% higher for GlutMed L vs. GlutMed R (*p* < 0.001), but not for the other muscle pairs (Table 5). However, SD CCIs was higher for all muscle pairs, 27% higher for TibAnt vs. MedGas; 34% higher for TibAnt vs. PerLong, and 38% higher for GlutMed L vs. GlutMed R (*p* < 0.001).

#### Influence of vision on CCI during the control tests

Within-subjects analyses revealed that vision did not significantly change mean and SD CCIs in the three muscle compositions investigated (Fig. 3; Table 6).

**Table 6:** Influence of vision on CCI during the control tests

CCI ^a, b^	*p*-value	EC/EO ^c^
Mean		
TibAnt vs. MedGas	0.350	0.98 (0.05)
TibAnt vs. PerLong	0.546	0.87 (0.04)
GlutMed L vs. GlutMed R	0.086	1.13 (0.09)
SD		
TibAnt vs. MedGas	0.399	0.89 (0.11)
TibAnt vs. PerLong	0.878	0.75 (0.08)
GlutMed L vs. GlutMed R	0.479	1.05 (0.09)

^a^Quotient values are presented as: group mean quotient (individual quotient SEM)

^b^A *p* < 0.05 was considered significant after Bonferroni correction

^b^Quotient values between the control tests with eyes closed /control tests with eyes open

### Correlation analysis

#### Correlation between postural stability vs. CCI during VR

The Spearman correlation analyses revealed for the mean CCI parameters one significant small positive association with postural stability parameters during VR in the total of 18 correlations performed between postural stability and mean CCI parameters (*p* = 0.024) (Table 7). For the SD CCI parameters, the evaluations revealed 8 significant small to moderate positive associations with postural stability parameters during VR in the 18 correlations performed (*p* ≤ 0.035).

**Table 7:** Correlation between postural stability vs. CCI during VR

Correlation postural stability vs. CCI	Mean^a^			SD^a^		
	TibAnt vs. MedGas	TibAnt vs. PerLong	GlutMed L vs. GlutMed *R*	TibAnt vs. MedGas	TibAnt vs. PerLong	GlutMed L vs. GlutMed *R*
Anteroposterior						
Total	0.142 [0.125]	**0.024 [0.195]**	0.234 [-0.102]	**< 0.001 [0.332]**	**< 0.001 [0.422]**	0.317 [0.085]
Low < 0.1 Hz	0.228 [0.103]	0.066 [0.159]	0.086 [-0.146]	**< 0.001 [0.290]**	**< 0.001 [0.373]**	0.551 [0.051]
High > 0.1 Hz	0.958 [-0.005]	0.198 [0.112]	0.266 [-0.095]	**0.035 [0.179]**	**< 0.001 [0.297]**	0.716 [0.031]
Lateral						
Total	0.979 [-0.002]	0.204 [0.111]	0.121 [-0.132]	0.310 [0.087]	**0.022 [0.198]**	0.594 [0.046]
Low < 0.1 Hz	0.251 [0.098]	0.160 [0.122]	0.209 [-0.107]	0.297 [0.089]	0.162 [0.121]	0.699 [-0.033]
High > 0.1 Hz	0.067 [-0.156]	0.918 [0.009]	0.172 [-0.116]	0.938 [0.007]	**0.031 [0.186]**	0.171 [0.117]

^a^p-values and [R-values]

Significant differences are shown in bold figures

#### Correlation between postural stability vs. CCI during control tests

The Spearman correlation analyses revealed for the mean CCI parameters no significant associations with postural stability parameters during the control tests in the total of 18 correlations performed (Table 8). For the SD CCI parameters, the evaluations revealed 2 significant small positive associations with stability parameters during the control tests in the 18 correlations performed (*p* ≤ 0.044).

**Table 8:** Correlation between postural stability vs. CCI during control tests

Correlation postural stability vs. CCI	Mean^a^			SD^a^		
	TibAnt vs. MedGas	TibAnt vs. PerLong	GlutMed L vs. GlutMed *R*	TibAnt vs. MedGas	TibAnt vs. PerLong	GlutMed L vs. GlutMed *R*
Anteroposterior						
Total	0.305 [-0.140]	0.450 [-0.105]	0.081 [-0.235]	0.247 [0.157]	0.135 [0.206]	0.348 [0.128]
Low < 0.1 Hz	0.552 [-0.081]	0.827 [-0.030]	0.138 [-0.201]	0.239 [0.160]	0.103 [0.224]	0.305 [0.140]
High > 0.1 Hz	0.652 [-0.062]	0.434 [-0.109]	0.642 [-0.063]	0.191 [0.178]	0.275 [0.151]	0.565 [0.079]
Lateral						
Total	0.906 [0.016]	0.382 [0.121]	0.542 [-0.083]	0.101 [0.221]	**0.042 [0.278]**	0.200 [0.174]
Low < 0.1 Hz	0.287 [0.145]	0.077 [0.243]	0.847 [0.026]	0.100 [0.222]	**0.044 [0.276]**	0.129 [0.205]
High > 0.1 Hz	0.337 [-0.131]	0.785 [-0.038]	0.239 [-0.160]	0.243 [0.159]	0.139 [0.204]	0.244 [0.158]

^a^p-values and [R-values]

Significant differences are shown in bold figures

## Discussion

The main aim of this study was to investigate whether postural control in young healthy adults, measured with force plate (stability measures) and EMG (muscle co-contractions, CCI), respond and adapt to repeated visual immersive VR. Another aim was to investigate if co-contraction of postural muscles was associated with stability measures during the tests. The results revealed that stability measures worsened significantly due to VR-stimulation, and they improved (decreased) in the anteroposterior direction due to repeated stimulation. In line with this, the mean and SD of the postural muscles’ CCI increased due to visual VR-stimulation and decreased as an adaptation over repeated VR-trials. This was shown for all three included muscle pairs, i.e., TibAnt vs. MedGas, TibAnt vs. PerLong, and GlutMed L vs. GlutMed R. The correlation analyses of the VR-sessions showed positive R-value correlations between stability measures and the CCI of the ankle muscle pairs, i.e. TibAnt vs. MedGas and TibAnt vs. PerLong. Here, positive R-values in the correlations represent that increased use of energy as recorded by the force platform was related to increased CCI. The strongest correlations were found between the stability measures in anteroposterior direction and the SD of the CCI. Interestingly, when comparing quiet stance with eyes open and eyes closed, i.e., the control conditions, there was a change in the stability measures but not in the CCI measures. Also, there were only small, mostly non-significant correlations between the stability measures and CCI during the control conditions.

These results show that immersive visual environments produced by VR devices in this study induce challenges to postural control that evoke both decreased postural stability (increased postural sway) and increased muscle activity co-contraction patterns. This was shown by the first VR session which produced significantly increased energy use for anteroposterior and lateral stability as well as the mean and SD CCI of the three muscle combinations, compared with the control test with eyes open. Moreover, the VR stimuli caused the association between postural stability and muscle activity co-contractions to become more prominent in a way that did not exist during the control tests. In the correlation analysis of the VR tests, all significant R-values had a positive sign representing that increased use of postural stability energy was associated with higher levels of muscle activity co-contractions. The findings of positive correlation associations between postural stability and co-contraction levels could be considered unexpected, as increased co-contractions generally are associated with increased dampening of movement that also should make the energy applied on a force platform from physical movements smaller (Franklin and Wolpert 2011). However, since this was a novel and unpredictable visual task that challenged balance, increased muscle activity could be an effect of the subjects adopting a more rigid single-link movement pattern in the human body’s multi-segmented domain to keep balance, i.e., a stiffening strategy. Typically, it requires less complex motor commands to control the stability of an object with fewer degrees of movement than an object where the entire stability is ensured from controlling multiple segments both individually and interrelated to each other (Franklin and Wolpert 2011). While this stiffening strategy can increase stability, it may be more energy consuming, fatiguing and less flexible and efficient over time.

The results showed that postural control mechanisms in young healthy adults could quickly reduce the effects of the novel visual challenges when given the opportunity to familiarize themselves with the situation over 5 repeated sessions performed 10 min apart. The adaptation resulted in both significantly less use of energy for postural stability and lower muscle activity co-contractions, indicating less stiffening strategy, possibly related to a more efficient use of feedback and feedforward control. This said, during VR session 5, postural stability in both anteroposterior and lateral directions and the SD CCI remained significantly greater than during the control tests with eyes open. Thus, training paradigms including repetitions may be able to produce substantial sensorimotor control changes that alter the influence of disruptive visual information, though it may require more than 5 sessions performed on one occasion to reach the sufficient effect of neutralization of the visual stimulation. In future research, it would be relevant to investigate the effects of several training occasions and to evaluate retention effects, and possible transfer effects to other balance tasks and daily activities.

### Direction and frequency specific effects

A VR environment may produce instabilities in anteroposterior and lateral directions of different magnitudes. The findings in this study suggest that the VR movie, the participants watched repeatedly, disturbed the postural stability more in anteroposterior direction than lateral directions over the trials. The fast corrective movements to maintain balance (i.e. high frequency energy > 0.1 Hz) were more affected by the VR stimuli than the smooth corrective changes of posture (i.e. low frequency energy < 0.1 Hz) (Kristinsdottir et al. 2001). The adaptation of postural stability in response to repeated VR trials reached significant effects only in the anteroposterior direction. Corresponding to the effects observed by the VR stimulation, the fast corrective changes of posture were more prone to adapt over the VR-trials than the smooth corrective movements to maintain balance (Kristinsdottir et al. 2001).

Increased co-contraction between TibAnt vs. MedGas is considered to predominantly increase the stability in anteroposterior direction whereas increased co-contractions between TibAnt vs. PerLong and GlutMed L vs. GlutMed R is considered to produce increased stability in lateral direction (Shumway-Cook 2008). However, the correlation analyses showed positive R-value correlations (i.e., increased stability measures as recorded by the force platform related to increased CCI) between anteroposterior stability measures and SD CCI for both TibAnt vs. MedGas and TibAnt vs. PerLong. Even though some significant correlations were found between lateral stability and the SD CCI of the muscle pair TibAnt vs. PerLong in both VR and control conditions, which support this direction specific effect of muscle activation, the p-values were close to non-significant, and thus, should be interpreted with caution. While these included muscle pairs that are considered important for postural stability in standing, to include more postural muscles at the ankle and other body parts could possibly improve the correlation analyses regarding direction specificity between stability measures and EMG.

### Association between postural stability and muscle activity

As mentioned above, the VR stimuli caused the association between postural stability and muscle co-contractions to become more prominent in a way that did not exist during the control tests. Correlations appeared most significantly between postural stability in anteroposterior directions and the SD CCIs of the ankle muscle pairs of TibAnt vs. MedGas and TibAnt vs. PerLong. This could indicate a couple of interesting facts. Firstly, the variation (SD) of the muscle activity co-contractions, rather than the mean values, correlates with the stability measures from the force plate during the VR-task. This may be explained by people often having their center of mass (COM) slightly anterior to the ankle joint, thereby largely controlling the stability of the ankle by the triceps surae muscles (including the MedGas), rather than a constant co-contraction between the plantar flexors (here represented by MedGas) and dorsal flexor (here represented by TibAnt). Secondly, the CCI around the ankles (TibAnt vs. MedGas, and TibAnt vs. PerLong), but not the hip (GlutMed L vs. GlutMed R), correlated with the stability measures. This could indicate that the participants used a single link ankle strategy during the VR-task. Both the COM’s position in relation to the ankle, and the possible use of single link ankle strategy, could be investigated in future studies by including motion capture to the measurement protocol.

During the control tests, only a few random significant correlations appeared between postural stability and CCI. Taken together, our findings suggest that when healthy young adults perform quiet stance tests, postural control applies a strategy that is manifested as seemingly erratic postural stability and muscle usage actions. However, when having to address more challenging stability situations, such as immersive visual environments from VR, postural control can change strategy to a more “active” and “rigid” mode where postural stability involves high energy actions and co-active muscle activations patterns to solve the task.

### Impact of distortive visual environments from virtual reality vs. receiving no visual information

Intuitively, one might assume that the most challenging visual condition is when no visual information is available, i.e., when standing with eyes closed. However, for example, the postural energy used within the high frequency range increased by 481% when the participants watched a challenging VR movie compared to the control tests with eyes open but only increased by 36% when the participants changed from standing with eyes open to standing with eyes closed. When studying the effects on muscle co-contractions, the mean CCI increased significantly, on average 53% for the three muscle combinations investigated, during the initial VR compared with eyes open condition. However, no significant change in CCI was observed between eyes closed vs. eyes open conditions. Hence, disrupted visual information can cause more severe challenges to postural control than a complete loss of visual information.

### Study limitations

Firstly, aims were limited to investigating the short-term effects of five repeated VR exposures on one occasion. It is possible that more repetitions and training occasions would lead to more complete adaptations. Other studies have shown that human posture can consolidate improvements of sensorimotor control from repeated sessions to balance perturbations, performed with 10 min intervals between each session (Tjernstrom et al. 2002; Tjernström et al. 2005), and still retain positive effects when exposed to the identical stability challenges 3 months later. The goal of training paradigms is to achieve long-term (retention) effects. Moreover, training effects should also be transferred to daily activities to have positive effects on the person’s life. These topics need to be addressed in future VR studies.

Secondly, this study was performed on healthy young adults. However, the individuals most commonly suffering from sensorimotor issues related to improper ability to reweight from flawed sensory information sources are older people. Moreover, many older people suffer from the fact that none of the alternative sensory systems to vision, e.g., the proprioceptive or vestibular systems, are unscathed from age-related decline, which might make some older people more vulnerable to immersive visual environments. This important topic will be addressed in a separate study.

Thirdly, only three muscle pairs were assessed with EMG to investigate muscle co-contraction due to VR stimulation. While the included muscles are considered vital postural muscles, it is still a simplified model for active control of postural stability. Regarding the plantar flexors, the soleus could have been an alternative muscle to measure. However, a previous study reported more activity in medial gastrocnemius among older adults compared to younger adults in a standing task. These differences were not shown for medial or lateral soleus (Dos Anjos et al. 2017). As we plan to explore the same experimental VR-setup also with other groups of participants, including healthy people of older age, we reasoned that MedGas would be most suitable to identify possible differences between groups.

## Conclusion

Immersive visual environments created by VR devices can produce marked stability challenges to postural control that evoke both decreased postural stability and altered muscle activation patterns (increased co-contractions). Moreover, the VR stimuli caused the association between postural stability and muscle activity co-contractions to become more prominent in a way that did not exist during the control tests. However, the postural control of young healthy adults can quickly reduce the effects of these novel visual challenges when given the opportunity to familiarize themselves with the situation over repeated sessions. The adaptation both resulted in significantly less use of energy for postural stability and less muscle co-contraction. Thus, VR stimuli might be utilized to enhance postural control fitness by increasing the resilience to disruptive information from key sensory sources.
